# A Randomized Phase 3 Study to Evaluate the Safety, Tolerability, and Immunogenicity of the Bivalent RSVpreF Vaccine in Older Adults in Korea

**DOI:** 10.3390/vaccines14090773

**Published:** 2026-09-03

**Authors:** Won Suk Choi, Karen Quan, James A. Baber, Anna Jaques, Karla Janse van Rensburg, Wen Li, Mark W. Cutler, Elena V. Kalinina, Annaliesa S. Anderson, Kena A. Swanson, Alejandra Gurtman, Iona Munjal

**Affiliations:** 1 Department of Internal Medicine, Korea University College of Medicine, Seoul 02708, Republic of Korea; 2 Pfizer Vaccines, Pfizer Australia, Sydney, NSW 2000, Australia; 3 Pfizer Vaccines, Pfizer UK, Marlow SL7 1HZ, Buckinghamshire, UK; 4 Pfizer Vaccines, Pfizer Inc., Collegeville, PA 19426, USA; 5 Pfizer Vaccines, Pfizer Inc., Pearl River, NY 10965, USA

**Keywords:** immunogenicity, Korea, older adults, respiratory syncytial virus, RSV vaccine

## Abstract

**Background/Objectives**: The bivalent RSVpreF vaccine is effective at preventing respiratory syncytial virus (RSV)-associated lower respiratory tract illness in adults. However, data regarding safety and immunogenicity of RSVpreF in Korean populations are limited. **Methods**: This was a phase 3, multicenter, placebo-controlled, randomized (2:1), double-blind study in Korean adults 60 years of age and older. Participants received a single 120-μg dose of RSVpreF or matching placebo. Safety endpoints included local reactions and systemic events through 7 days and adverse events (AEs) through 1 month after vaccination, and serious AEs (SAEs) throughout the study. RSV-A and RSV-B serum 50% neutralizing geometric mean titers (GMTs) and geometric mean fold rises (GMFRs) were obtained 1 month after vaccination. **Results**: Overall, 377 participants received study intervention (RSVpreF, *n* = 251; placebo, *n* = 126). Most local reactions and all systemic events were of mild or moderate severity. Injection-site pain was the most frequently reported local reaction (RSVpreF, 12.4%; placebo, 3.2%). The most frequently reported systemic events were fatigue (RSVpreF, 23.1%; placebo, 26.2%) and muscle pain (RSVpreF, 15.5%; placebo, 9.5%). AEs through 1 month after vaccination were infrequent (RSVpreF, 3.6%; placebo, 0.8%); none were considered vaccine related by the investigator. RSV-A and RSV-B neutralizing GMTs increased 1 month after RSVpreF, with GMFRs (95% CIs) from before to 1 month after vaccination of 9.5 (8.51–10.67) for RSV-A and 8.3 (7.37–9.39) for RSV-B. **Conclusions**: In older Korean adults, RSVpreF had an acceptable safety and tolerability profile and elicited robust RSV neutralizing responses 1 month after vaccination consistent with pivotal phase 3 efficacy trial results. ClincalTrials.gov Identifier: NCT06593587 (date of registration: 9 September 2024).

## 1. Introduction

Respiratory syncytial virus (RSV) is a clinically significant respiratory pathogen in individuals of all ages and can result in severe lower respiratory tract illness (LRTI) in infants, older adults, and those with health conditions that put them at increased risk of severe RSV and complications [1,2,3]. Adults 60 years and older are at increased risk of severe RSV infection [4], which can exacerbate underlying comorbid conditions, such as chronic obstructive pulmonary disease [5]. RSV illness presents a substantial global burden among older adults, leading to millions of cases, hundreds of thousands of hospitalizations, and tens of thousands of deaths annually [6,7,8]. In addition, beyond its acute clinical consequences, RSV may also contribute to functional decline in older adults [9]. Data on the epidemiology of RSV show that it is a common causative pathogen in acute respiratory illness in Korean adults [10,11,12]. However, the true burden of RSV in older adults is likely underestimated globally because of a lack of routine testing and inadequate standardized surveillance [8]. Additionally, as populations continue to age and the number of older adults increases, the global burden of RSV disease and its associated complications is expected to grow [13].

After natural RSV infection, immunity is short-lived, and repeat infections are possible [14,15]; vaccination is therefore an important tool to help protect against severe RSV-associated disease, especially in the elderly or those with comorbidities [16]. The bivalent RSV prefusion F protein vaccine, RSVpreF (Abrysvo^®^ (respiratory syncytial virus vaccine, unadjuvanted); Pfizer Inc., New York, NY, USA), is well tolerated in older adults and is efficacious in preventing RSV-associated LRTI [17,18]. RSVpreF is also well tolerated during pregnancy, and maternal vaccination has been effective in preventing LRTI attributed to RSV in infants from birth through 6 months after birth [19]. RSVpreF has received marketing authorization by the US Food and Drug Administration and the European Commission, as well as other regulatory agencies [20,21]. However, there are limited data on the safety and immunogenicity of the RSVpreF vaccine in the Korean population. A monovalent prefusion F vaccine (Arexvy^®^ (respiratory syncytial virus vaccine, adjuvanted); GlaxoSmithKline, Durham, NC, USA) is approved in the Republic of Korea for prevention of RSV-associated LRTI in older adults [22] based on the results of a phase 3 trial that included participants from Korea [23].

The objective of this study was to describe the safety, tolerability, and immunogenicity of the bivalent RSVpreF vaccine in Korean adults 60 years and older.

## 2. Methods

### 2.1. Study Design and Participants

This was a phase 3, randomized, double-blind, placebo-controlled study conducted between 7 October 2024 and 3 February 2025 (ClinicalTrials.gov Identifier: NCT06593587; date of registration: 9 September 2024). This study is reported here in accordance with Consolidated Standards of Reporting Trials (CONSORT) 2025 guidelines (Table S1).

Study participants were enrolled at 16 clinical trial sites in the Republic of Korea. Participants were randomized in a 2:1 ratio to receive a single injection of either RSVpreF 120 μg (lot number 21-DP-00546) or placebo (lot number 21-DP-00729) in the deltoid muscle. The placebo consisted of excipients matched to those used in the RSVpreF formulation, minus the active ingredients, with a matched physical appearance. Participants and study site personnel were blinded to vaccine allocation. Randomization was conducted using interactive response technology and participants were stratified by three age groups: 60 through 69, 70 through 79, and ≥80 years of age. Participants were followed up for 2 months after study vaccination.

The study was carried out in compliance with the approved protocol and with consensus ethical standards based on international guidance, including the Declaration of Helsinki, the International Council for Harmonisation Good Clinical Practice guidelines, and all relevant laws and regulations, inclusive of those governing data privacy. Before the study could begin, the investigator submitted the protocol, informed consent materials, and all other pertinent documents to the institutional review board at his or her site, where they were evaluated and approved. Before being enrolled in the study, each participant was required to provide written informed consent.

The study included healthy participants (including those with preexisting stable disease) 60 years and older. Male participants with reproductive potential agreed to use a highly effective contraceptive method through 28 days after receipt of the study intervention. Participants of female sex were to be of nonchildbearing potential. Individuals were excluded if they had confirmed RSV infection within 180 days of enrollment; bleeding diathesis or a condition associated with prolonged bleeding that would contraindicate intramuscular injection; a history of any subtype of Guillain−Barré syndrome (GBS); a history of severe adverse reaction associated with a vaccine or severe allergic reaction to any component of the study interventions or any related vaccine; a serious chronic disorder that, in the investigator’s opinion, would exclude the individual from participating in the study; or individuals who were immunocompromised or experiencing immunodeficiency. Additionally, individuals were excluded if they had been previously vaccinated with any RSV vaccine, received high-dose chronic systemic corticosteroids for ≥14 days from 28 days before study intervention, received other immunosuppressive medications or blood or plasma products within 60 days before study intervention, or had participated in other studies involving investigational drugs or vaccines within 6 months before study intervention. Participants could receive any nonlive or recombinant vaccine more than 14 days before or any live vaccine more than 28 days before administration of study intervention. If a participant who was otherwise eligible had a condition meeting temporary delay criteria (including a current febrile illness (temperature ≥ 38.0 °C) or other acute illness within 48 h before study intervention), randomization and vaccination could be delayed until that condition resolved or delay criteria were no longer met.

### 2.2. Objectives and Endpoints

The primary safety objective was to describe tolerability and safety of the RSVpreF vaccine in Korean adults 60 years and older. Participants were observed for immediate reactions for at least 30 min after receiving study intervention. Prespecified reactogenicity events (local reactions and systemic events) were prospectively collected by participants using an electronic diary. Reports of local reactions (redness, swelling, and injection-site pain) and systemic events (fever, fatigue, headache, vomiting, nausea, diarrhea, muscle pain, and joint pain) were collected for 7 days after receiving study intervention (i.e., Day 1 through Day 7 after vaccination), or longer if symptoms were ongoing. Local reactions and systemic events were graded on a severity scale (Appendix A Table A1). Additionally, prespecified reactogenicity events were categorized as those protocol-specified local reactions and systemic events that were immediate events (i.e., onset within 30 min of vaccination), reactogenicity events leading to withdrawal, medically attended reactogenicity events (i.e., grade 4 reactogenicity events), and reactogenicity events that were also considered serious adverse events (SAEs). Nonserious adverse events (AEs) were collected through 1 month after study vaccine administration, while SAEs and newly diagnosed chronic medical conditions (NDCMCs) were collected through 2 months after vaccination (i.e., through the study). AEs of special interest (AESIs; i.e., a diagnosis of GBS, acute polyneuropathy without an underlying etiology, or atrial fibrillation) were collected throughout the study.

The primary immunogenicity objective was to describe the immune responses to RSV-A and RSV-B elicited by RSVpreF vaccination in Korean adults 60 years and older. Blood samples were obtained before and 1 month after vaccination and assayed for neutralizing antibody titers against both RSV subgroups (i.e., RSV-A and RSV-B) using methodology described previously [24]. Primary immunogenicity endpoints were geometric mean titers (GMTs) and geometric mean fold rises (GMFRs) of RSV-A and RSV-B neutralizing titers. The percentage of participants with a seroresponse 1 month after vaccination (i.e., RSV neutralizing titers ≥ 4 × lower limit of quantitation (LLOQ) if the measurements before vaccination were <LLOQ or a ≥4-fold rise from baseline if the measurements before vaccination were ≥LLOQ) was a secondary immunogenicity endpoint.

### 2.3. Statistical Analysis

This was a descriptive study with no formal hypothesis testing. All participants who received study intervention comprised the safety population. Descriptive statistics were provided for each safety endpoint by intervention group. The participants who met eligibility requirements, received the study intervention as randomized, had ≥1 valid and determinate assay result 1 month after receiving study intervention, and had no major protocol deviations from the time of receiving study intervention through the blood draw that occurred 1 month later comprised the evaluable immunogenicity population. It was expected that 240 participants would receive the RSVpreF vaccine and assuming about a 10% nonevaluable rate, approximately 210 of these participants would be assessed for the immunogenicity endpoints. Immunogenicity data were summarized as RSV-A and RSV-B neutralizing GMTs before and 1 month after vaccination and GMFRs from before to 1 month after vaccination, with associated 95% CIs, by intervention group. GMTs were calculated by exponentiating the mean and GMFRs by exponentiating the mean difference of the logarithmically transformed neutralizing titers, with the associated 95% CIs determined using the Student’s *t* distribution. The percentage of participants with seroresponse at 1 month after vaccination was calculated together with the associated two-sided 95% CIs, which was determined using the Clopper–Pearson method.

## 3. Results

### 3.1. Participants

**Figure 1:**
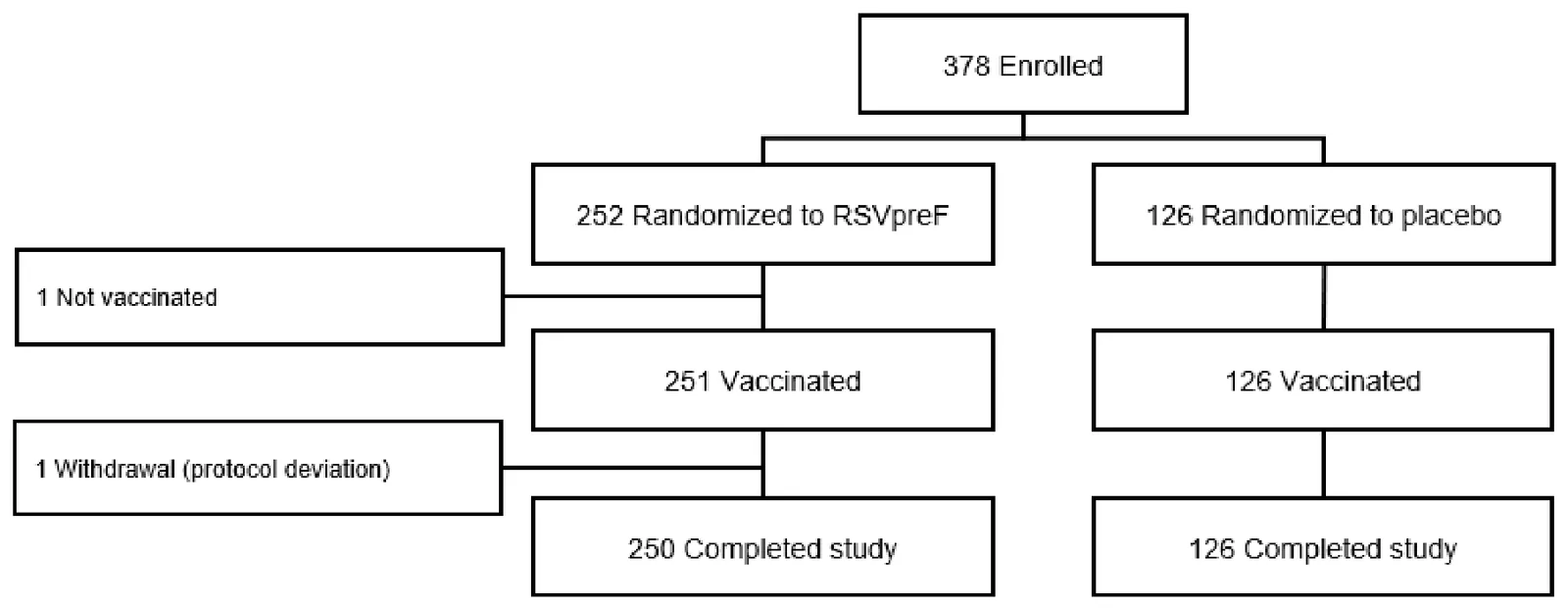
Study disposition. RSVpreF, respiratory syncytial virus prefusion F protein vaccine.

Overall, 378 participants were randomized 2:1 to receive RSVpreF or placebo. Of these, 377 participants (99.7%) were vaccinated (251 participants received RSVpreF and 126 participants received placebo; Figure 1). Most participants (99.5%) completed the study; one participant in the RSVpreF group withdrew because of a protocol deviation.

Demographic characteristics were balanced between groups. All participants were Asian, 64.7% (244/377) were of female sex, and the median age at vaccination was 66 years (range, 60–89 years; Table 1). The majority of participants were 60 to 69 years of age (72.1% (272/377)); 26.3% (99/377) were 70 to 79 years of age, and 1.6% (6/377) were 80 years and older. More than a quarter of participants had at least one significant prespecified medical condition considered to increase the risk of severe RSV-associated illness (27.1% (102/377)), most commonly diabetes mellitus (18.3% (69/377)), current tobacco use (6.4% (24/377)), and heart disease (i.e., congestive heart failure or other heart disease; 5.3% (20/377)).

### 3.2. Safety

**Figure 2:**
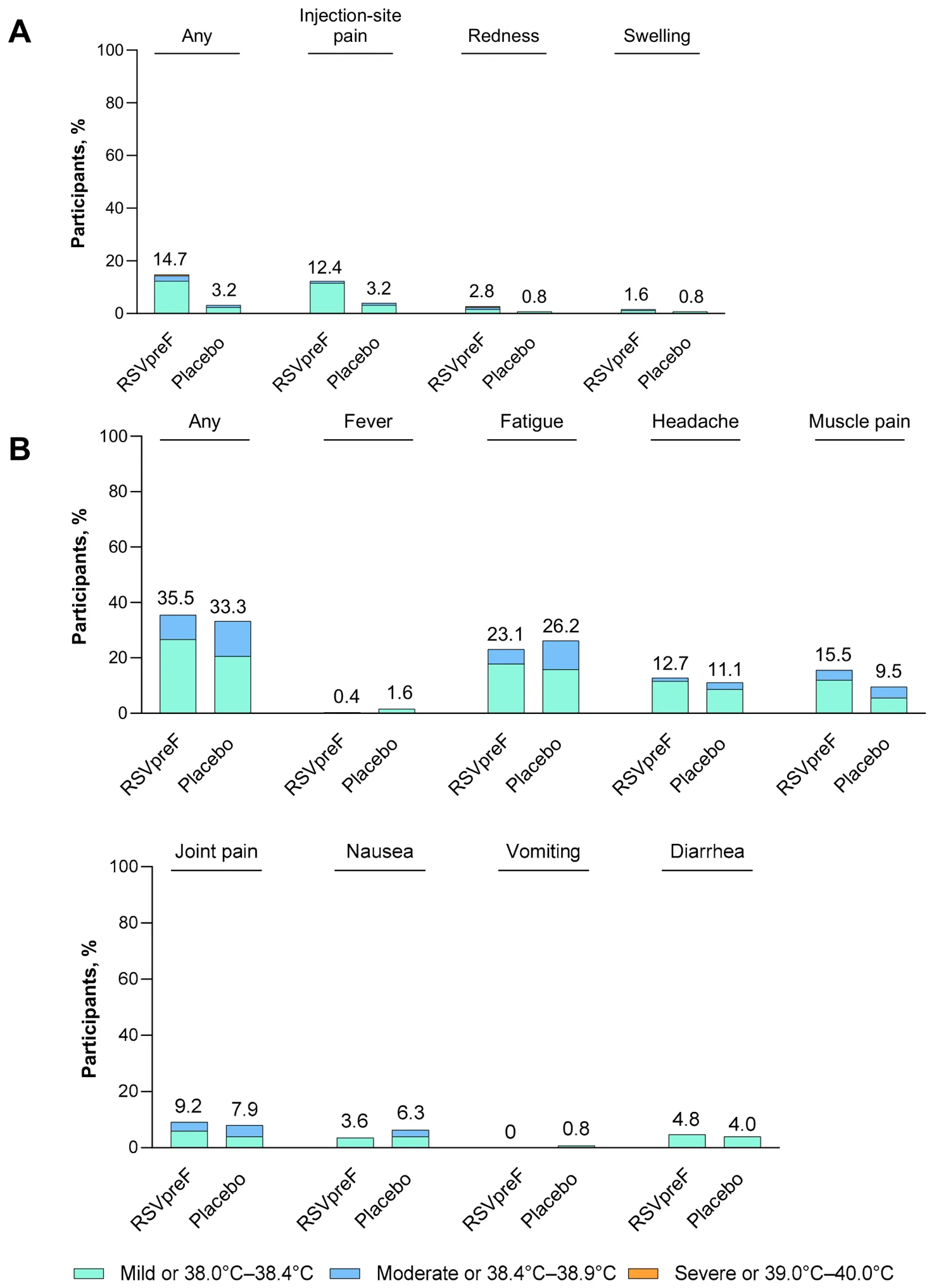
(**A**) Local reactions and (**B**) systemic events by maximum severity reported within 7 days after vaccination. Data are for the safety population (all participants who received study intervention). The numbers above the bars are the percentages of participants overall with a local reaction or systemic event of any severity. The severity scale for local reactions and systemic events is shown in Appendix A Table A1. The number of participants was as follows: RSVpreF, N = 251; placebo, N = 126. RSVpreF, respiratory syncytial virus prefusion F protein vaccine.

All 377 participants who received RSVpreF or placebo were included in the safety population. Local reactions and systemic events with onset ≤7 days after vaccination are shown in Figure 2A. The percentage of participants reporting local reactions was 14.7% (37/251) in the RSVpreF group and 3.2% (4/126) in the placebo group, with injection-site pain the most frequently reported local reaction (RSVpreF, 12.4% (31/251); placebo, 3.2% (4/126)). Most local reactions were mild or moderate in severity, with severe redness reported in one participant (0.4%) in the RSVpreF group and no grade 4 local reactions were reported. Median onset of local reactions in the RSVpreF group was between Day 2 and 6 after vaccination (on Day 1), and median resolution was 1 to 1.5 days after onset. The percentage of participants who reported systemic events was generally similar in the RSVpreF (35.5% (89/251)) and placebo (33.3% (42/126)) groups (Figure 2B). The most frequently reported systemic events were fatigue (RSVpreF, 23.1% (58/251); placebo, 26.2% (33/126)) and muscle pain (RSVpreF, 15.5% (39/251); placebo, 9.5% (12/126)). All systemic events were mild or moderate in severity; no severe or grade 4 systemic events were reported. Few events of fever were reported (≤1.6% in either group); all fever events were mild or moderate and no severe (>38.9 °C−40.0 °C) or grade 4 fevers (>40.0 °C) were reported. Median onset of systemic events in the RSVpreF group was Day 2 to Day 5 after vaccination, and median resolution was 1 to 4 days after onset. No reactogenicity events were immediate events, events leading to withdrawal, medically attended events, or SAEs.

Adverse events through 1 month after study vaccination were infrequent and occurred in 3.6% of participants (9/251) in the RSVpreF group and 0.8% of participants (1/126) in the placebo group; none were assessed as related to study intervention by the investigator (Table 2). All AEs were mild or moderate in severity; no immediate or life-threatening AEs were reported. SAEs occurring throughout the study were reported in 0.8% of participants (2/251) in the RSVpreF group (enteritis and asthma) and in 0.8% of participants (1/126) in the placebo group (retinal detachment); all SAEs were considered to be unrelated to study intervention. Three of 251 participants in the RSVpreF group (1.2%) reported NDCMCs (hyperlipidemia and hypertension in one participant; type 2 diabetes mellitus and osteoporosis in one participant each); none were considered to be related to study intervention. There were no AESIs, withdrawals due to AEs, or deaths during the study.

### 3.3. Immunogenicity

**Figure 3:**
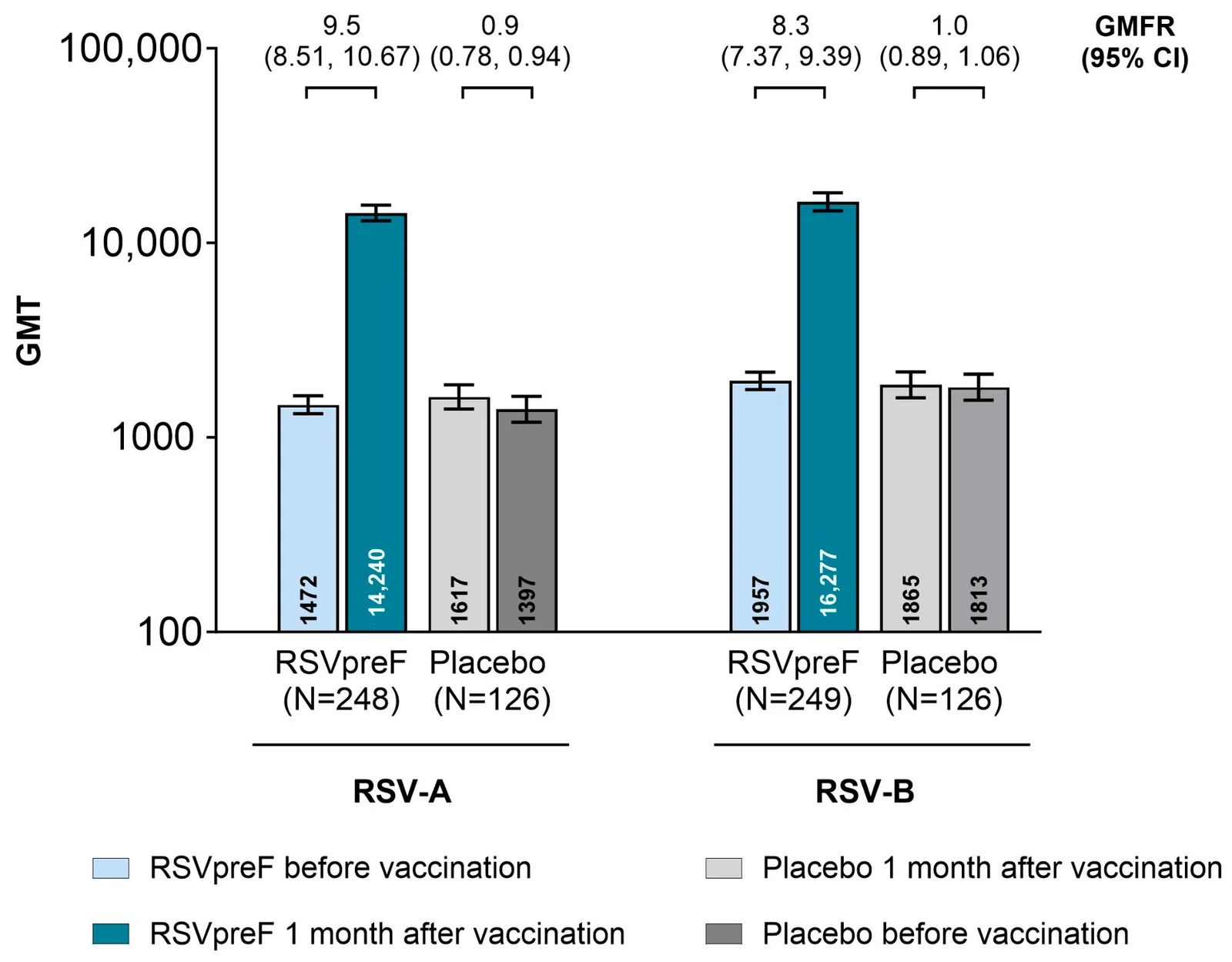
RSV-A and RSV-B 50% neutralizing titers before and 1 month after vaccination and GMFRs from before to 1 month after vaccination. Data are for the evaluable immunogenicity population. Values within the bars are GMTs and error bars are 95% CIs. Values above the bars are the GMFRs and 95% CIs from before to 1 month after vaccination. The LLOQs for each 50% neutralization titer were 242 (RSV-A) and 99 (RSV-B). When calculating a fold rise, if assay results were <LLOQ, the assay results were converted to 0.5 × LLOQ, except when the prevaccination assay result was <LLOQ while the postvaccination result was ≥LLOQ, in which case the prevaccination value was set to the LLOQ. GMTs and GMFRs were calculated by exponentiating the mean logarithm of the titers or the fold rises with corresponding 95% CIs based on the Student’s *t* distribution. GMT, geometric mean titer; GMFR, geometric mean fold rise; LLOQ, lower limit of quantitation; RSV, respiratory syncytial virus; RSVpreF, respiratory syncytial virus prefusion F protein vaccine.

**Figure A1:**
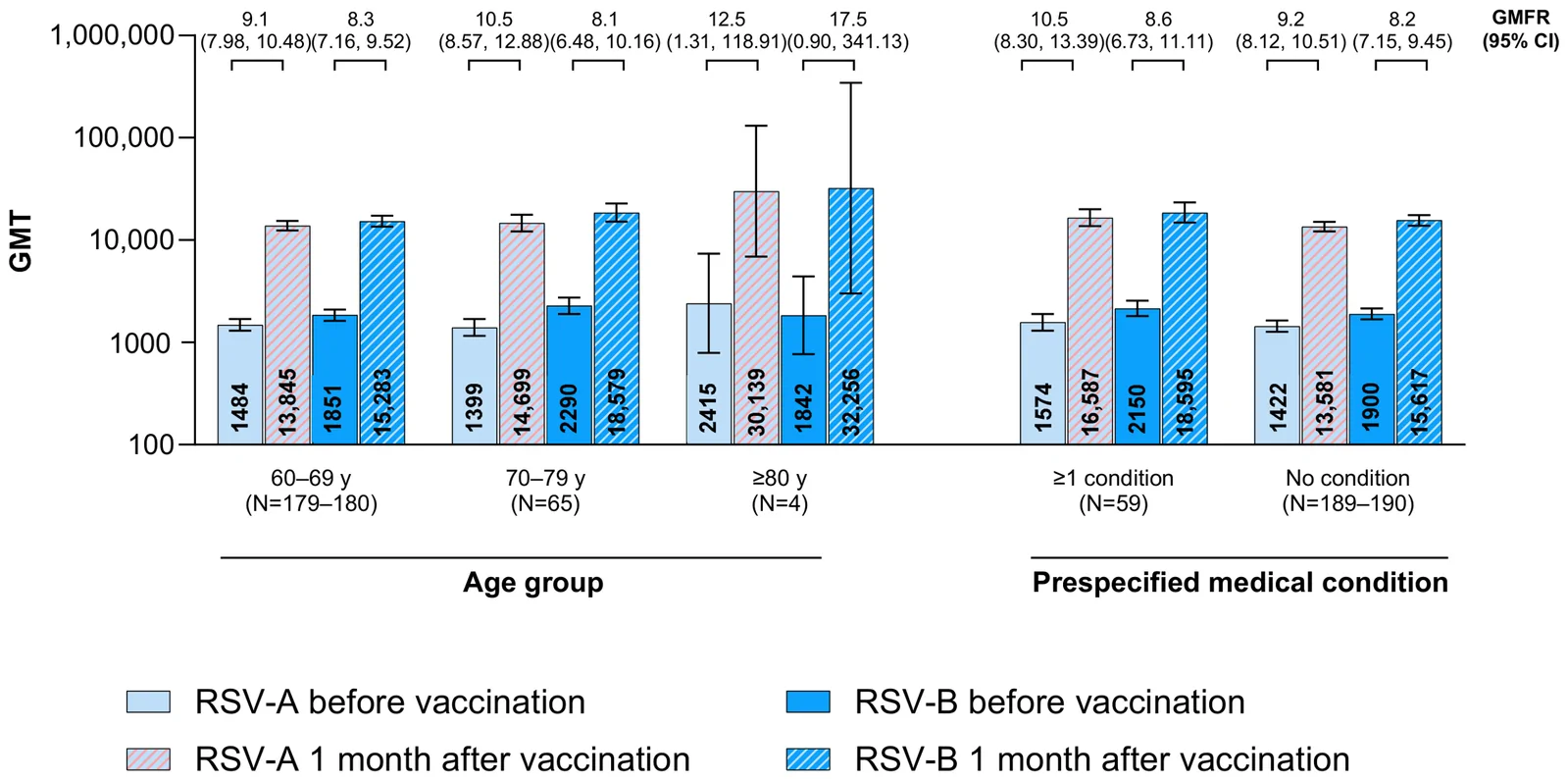
RSV-A and RSV-B 50% neutralizing titers before and 1 month after RSVpreF vaccination and GMFRs from before to 1 month after vaccination. Data are for the evaluable immunogenicity population. Values within the bars are GMTs and error bars are 95% CIs. Values above the bars are the GMFRs and 95% CIs from before to 1 month after vaccination. The LLOQs for each 50% neutralization titer were 242 (RSV-A) and 99 (RSV-B). When calculating a fold rise, if assay results were <LLOQ, the assay results were converted to 0.5 × LLOQ, except when the prevaccination assay result was <LLOQ while the postvaccination result was ≥LLOQ, in which case the prevaccination value was set to the LLOQ. GMTs and GMFRs were calculated by exponentiating the mean logarithm of the titers or the fold rises with corresponding 95% CIs based on the Student’s *t* distribution. Prespecified significant medical conditions included heart disease, lung disease, asthma, diabetes mellitus, liver disease, renal disease, and current tobacco use. GMT, geometric mean titer; GMFR, geometric mean fold rise; LLOQ, lower limit of quantitation; RSV, respiratory syncytial virus; RSVpreF, respiratory syncytial virus prefusion F protein vaccine.

The evaluable immunogenicity population included 249 of the 252 participants from the RSVpreF group (one participant was not vaccinated and two participants did not undergo the 1-month blood draw) and all 126 participants in the placebo group. In the evaluable immunogenicity population, neutralizing GMTs for RSV-A and RSV-B increased substantially at 1 month after the RSVpreF vaccination (Figure 3). GMFRs (95% CI) after RSVpreF vaccination were 9.5 (8.51, 10.67) for RSV-A and 8.3 (7.37, 9.39) for RSV-B; corresponding GMFRs in the placebo group for RSV-A and RSV-B were 0.9 and 1.0. When analyzed by age group, and with or without prespecified significant medical condition(s), RSV-A and RSV-B GMTs and GMFRs were consistent with those observed in the overall evaluable immunogenicity population (Appendix A Figure A1).

**Figure 4:**
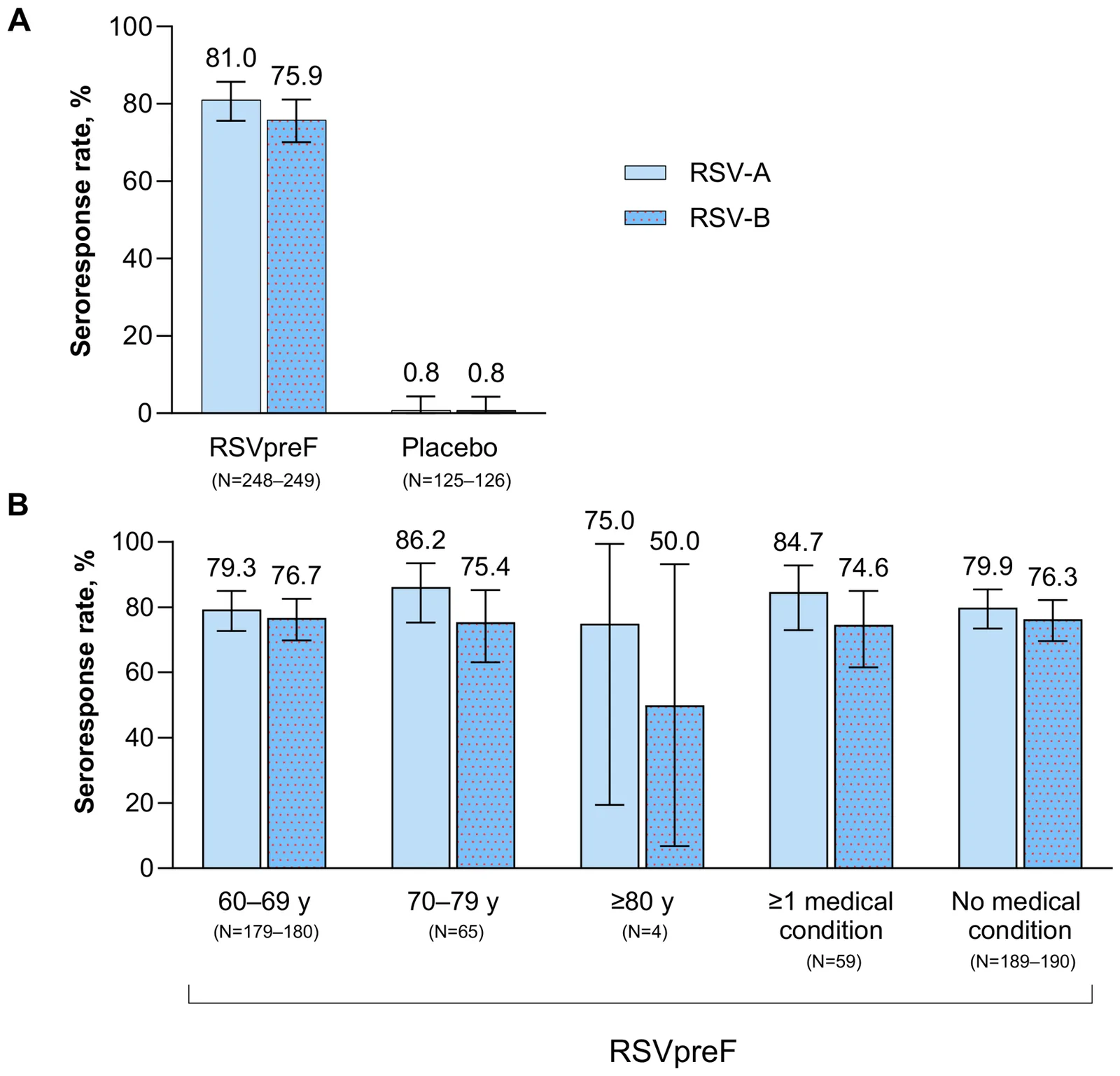
The percentage of participants achieving RSV-A and RSV-B 50% neutralizing titer seroresponse at 1 month after vaccination (**A**) overall and (**B**) by age group and with or without prespecified significant medical condition(s). Data are for the evaluable immunogenicity population. The LLOQs for each 50% neutralization titer were 242 (RSV-A) and 99 (RSV-B). Seroresponse was defined as achieving a ≥4-fold rise from before vaccination if the baseline measurement was >LLOQ. If the baseline measurement was <LLOQ, a postvaccination assay result ≥4 × LLOQ was considered a seroresponse. Exact two-sided 95% CIs were calculated using the Clopper–Pearson method. Prespecified significant medical conditions included heart disease, lung disease, asthma, diabetes mellitus, liver disease, renal disease, and current tobacco use. LLOQ, lower limit of quantitation; RSV, respiratory syncytial virus; RSVpreF, respiratory syncytial virus prefusion F protein vaccine.

In the evaluable immunogenicity population, the percentages of participants in the RSVpreF group with seroresponse at 1 month after vaccination were 81.0% (95% CI: 75.6, 85.7) for RSV-A and 75.9% (95% CI: 70.1, 81.1) for RSV-B serum neutralizing titers (Figure 4A). Only one participant (0.8%) in the placebo group met the seroresponse threshold for RSV-A and RSV-B. The percentages of participants with seroresponse were consistent across age groups and with or without prespecified significant medical condition(s) (Figure 4B).

## 4. Discussion

Respiratory syncytial virus-associated hospitalization and mortality burden in older adults is similar to that of seasonal influenza, highlighting the need for vaccination in this population [25]. The healthcare resource utilization burden is high for RSV-associated disease, particularly among older adults [26]. This burden of RSV on healthcare systems is particularly relevant in countries with large elderly populations, including Korea, where adults 65 years and older are predicted to exceed 20% of the population in 2025 [27]. In addition to hospitalization, RSV infection in older adults can lead to prolonged recovery and functional deterioration, with the greatest impact often observed among individuals with underlying chronic medical conditions who are at increased risk of severe disease [9,13]. As populations continue to age, the clinical and economic burden associated with RSV is expected to increase, placing further pressure on healthcare resources [13]. Effective vaccination strategies therefore have the potential to not only reduce RSV-related morbidity and healthcare utilization but also support healthy ageing and lessen the broader societal impact of RSV disease.

In this phase 3 study, RSVpreF was safe and well tolerated in Korean adults 60 years and older. Most reactogenicity events within 7 days after vaccination were mild to moderate in severity and were transient. This favorable reactogenicity profile appears to be consistent with that of the monovalent adjuvanted prefusion F vaccine already approved in Korea [28]. There were no immediate AEs, no AEs or SAEs were considered related to study intervention by the investigator, and there were no AESIs or AEs leading to withdrawal from the study. Within 1 month of vaccination, AEs were reported in 3.5% of participants in the RSVpreF group and 0.8% in the placebo group. Although these rates of AEs were lower than those observed in previous RSV vaccine clinical trials over a similar follow-up period [28,29], direct comparisons across studies should be interpreted with caution because of differences in study populations, trial designs, and settings. The low incidence of AEs observed in this study in both the RSVpreF and placebo groups may reflect the relatively healthy study population and small sample size. Overall, this favorable safety and tolerability profile is consistent with extensive data observed in previous phase 3 clinical trials of RSVpreF, including the global RENOIR study in adults 60 years and older from countries including the United States, Canada, Europe, and Japan [17], pregnant individuals ≤ 49 years of age [19], adults 18 to <60 years of age at high risk of severe RSV disease [30], and adults 18 years and older who are immunocompromised [31].

Although a correlate of protection against RSV disease has not been established, higher serum neutralizing antibodies are associated with reduced risk of RSV disease [32]. In this study, RSVpreF elicited robust neutralizing responses to both RSV-A and RSV-B in Korean adults 60 years and older, with GMFRs of ≥8.3 from before to 1 month after vaccination and ≥75.9% of participants meeting seroresponse thresholds 1 month after vaccination. Previous RSVpreF adult clinical trials have also reported robust neutralizing responses [17,19,30]. In the RENOIR trial in adults 60 years and older, RSV neutralizing antibody titers aligned with durable vaccine efficacy against RSV-associated LRTI over two full RSV seasons [17]. In the current study, neutralizing titers and seroresponses were consistent when analyzed by age subgroup (60–69, 70–79, and ≥80 years of age) and by the presence or absence of prespecified significant medical conditions (heart disease, lung disease, asthma, diabetes mellitus, liver disease, renal disease, and current tobacco use); this is in line with the RENOIR study results in which immune responses were observed regardless of age or high-risk subgroup [17]. Vaccine effectiveness of RSVpreF against RSV-associated illness among older adults in the United States has been confirmed by real-world evidence [18]. We anticipate that results from the current study suggest potential for RSVpreF to provide clinically meaningful protection against RSV disease in Korean adults, which was recently licensed in Korea for various indications, including for the prevention of LRTI caused by RSV in individuals 60 years and older [33].

Strengths of this study include the randomized, double-blind design and inclusion of a placebo comparator. Study limitations include descriptive statistical analyses and the inclusion of participants only from Korea, limiting generalizability to populations of other countries, and the small sample size of some study subgroups. The safety follow-up period was limited to 2 months after vaccination, and therefore longer-term safety outcomes were not evaluated. Efficacy and durability of immune responses were not assessed.

## 5. Conclusions

The RSVpreF vaccine had an acceptable safety and tolerability profile, and at 1 month after vaccination, robust RSV-A and RSV-B neutralizing responses were elicited in adults 60 years and older in Korea.

## Figures and Tables

**Table 1:** Demographic and baseline clinical characteristics of study participants.

Characteristic	RSVpreF (N = 251)	Placebo (N = 126)	Total (N = 377)
Sex, *n* (%)			
Male	83 (33.1)	50 (39.7)	133 (35.3)
Female	168 (66.9)	76 (60.3)	244 (64.7)
Asian race, *n* (%)	251 (100.0)	126 (100.0)	377 (100.0)
Non-Hispanic/non-Latino, *n* (%)	251 (100.0)	126 (100.0)	377 (100.0)
Age at vaccination			
Mean (standard deviation), years	67.3 (5.18)	67.3 (5.19)	67.3 (5.17)
Median (range), years	66.0 (60, 89)	67.0 (60, 83)	66.0 (60, 89)
60–69 years, *n* (%)	181 (72.1)	91 (72.2)	272 (72.1)
70–79 years, *n* (%)	66 (26.3)	33 (26.2)	99 (26.3)
≥80 years, *n* (%)	4 (1.6)	2 (1.6)	6 (1.6)
≥1 prespecified significant medical condition, *n* (%)	59 (23.5)	43 (34.1)	102 (27.1)
Current tobacco use	10 (4.0)	14 (11.1)	24 (6.4)
Diabetes mellitus	42 (16.7)	27 (21.4)	69 (18.3)
Lung disease ^a^	2 (0.8)	2 (1.6)	4 (1.1)
Heart disease ^b^	12 (4.8)	8 (6.3)	20 (5.3)
Liver disease	0	3 (2.4)	3 (0.8)
Renal disease	2 (0.8)	0	2 (0.5)
≥1 chronic cardiopulmonary condition, *n* (%)	1 (0.4)	2 (1.6)	3 (0.8)
Asthma	1 (0.4)	0	1 (0.3)
COPD	1 (0.4)	1 (0.8)	2 (0.5)
CHF	0	1 (0.8)	1 (0.3)
No prespecified significant medical condition, *n* (%)	192 (76.5)	83 (65.9)	275 (72.9)

CHF, chronic heart failure; COPD, chronic obstructive pulmonary disease; RSVpreF, respiratory syncytial virus prefusion F protein vaccine. Data are for the safety population (all participants who received study intervention). ^a^ Includes COPD and other lung disease. ^b^ Includes CHF and other heart disease.

**Table 2:** Adverse events.

Adverse Event	RSVpreF (N = 251)	Placebo (N = 126)
Through 1 month after vaccination		
Any AE	9 (3.6)	1 (0.8)
Related AE	0	0
Immediate ^a^	0	0
Severe	0	0
Life-threatening	0	0
Throughout the study		
Any SAE	2 (0.8)	1 (0.8)
Related SAE	0	0
Any NDCMC	3 (1.2)	0
Related NDCMC	0	0
Any AESI	0	0
AE leading to withdrawal	0	0
Deaths	0	0

Data are presented as *n* (%). AE, adverse event; AESI, adverse event of special interest; NDCMC, newly diagnosed chronic medical condition; RSVpreF, respiratory syncytial virus prefusion F protein vaccine; SAE, serious adverse event. Data are for the safety population (all participants who received study intervention). ^a^ Onset within 30 min after vaccination.

## Data Availability

Upon request, and subject to review, Pfizer will provide the data that support the findings of this study. Subject to certain criteria, conditions, and exceptions, Pfizer may also provide access to the related individual de-identified participant data. See https://www.pfizer.com/science/clinical-trials/trial-data-and-results (1 June 2026) for more information.

## Supplementary Materials

The following supporting information can be downloaded at: https://www.mdpi.com/article/10.3390/vaccines14090773/s1, Table S1: CONSORT 2025 checklist.

## Abbreviations

The following abbreviations are used in this manuscript:

AE	Adverse event
AESI	Adverse event of special interest
CONSORT	Consolidated Standards of Reporting Trials
GBS	Guillain–Barré syndrome
GMFR	Geometric mean fold rise
GMT	Geometric mean titer
LLOQ	Lower limit of quantitation
LRTI	Lower respiratory tract illness
NDCMC	Newly diagnosed chronic medical condition
RSV	Respiratory syncytial virus
RSVpreF	Respiratory syncytial virus prefusion F protein vaccine
SAE	Serious adverse event

## Appendix A

**Table A1:** Severity scale for local reactions and systemic events.

	Mild	Moderate	Severe	Grade 4
**Local reaction**				
Redness	>2.0–5.0 cm (5–10 measuring device units)	>5.0–10.0 cm (11–20 measuring device units)	>10 cm (>20 measuring device units)	Necrosis or exfoliative dermatitis
Swelling	>2.0–5.0 cm (5–10 measuring device units)	>5.0–10.0 cm (11–20 measuring device units)	>10 cm (>20 measuring device units)	Necrosis
Injection site pain	Does not interfere with activity	Interferes with activity	Prevents daily activity	Emergency department visit or hospitalization for severe injection site pain
**Systemic event**				
Fatigue	Does not interfere with activity	Some interference with activity	Prevents daily routine activity	Emergency department visit or hospitalization for severe fatigue
Headache	Does not interfere with activity	Some interference with activity	Prevents daily routine activity	Emergency department visit or hospitalization for severe headache
Vomiting	1–2 times in 24 h	>2 times in 24 h	Requires intravenous hydration	Emergency department visit or hospitalization for severe vomiting
Nausea	Does not interfere with activity	Some interference with activity	Prevents daily routine activity	Emergency department visit or hospitalization for severe nausea
Diarrhea	2–3 loose stools in 24 h	4–5 loose stools in 24 h	≥6 loose stools in 24 h	Emergency department visit or hospitalization for severe diarrhea
Muscle pain	Does not interfere with activity	Some interference with activity	Prevents daily routine activity	Emergency department visit or hospitalization for severe muscle pain
Joint pain	Does not interfere with activity	Some interference with activity	Prevents daily routine activity	Emergency department visit or hospitalization for severe joint pain
Fever	38.0–38.4 °C	>38.4–38.9 °C	>38.9–40.0 °C	>40.0 °C
